# Insulin-like growth factor 1/Child-Turcotte-Pugh composite score as a predictor of treatment outcomes in patients with advanced hepatocellular carcinoma treated with sorafenib

**DOI:** 10.18632/oncotarget.27924

**Published:** 2021-04-13

**Authors:** Yehia I. Mohamed, Sunyoung Lee, Lianchun Xiao, Manal M. Hassan, Aliya Qayyum, Rikita Hiatia, Roberto Carmagnani Pestana, Abedul Haque, Bhawana George, Asif Rashid, Dan G. Duda, Hesham Elghazaly, Robert A. Wolff, Jeffrey S. Morris, James Yao, Hesham M. Amin, Ahmed O. Kaseb

**Affiliations:** ^1^Department of Gastrointestinal Medical Oncology, The University of Texas MD Anderson Cancer Center, Houston, TX, USA; ^2^Department of Biostatistics, The University of Texas MD Anderson Cancer Center, Houston, TX, USA; ^3^Department of Epidemiology, The University of Texas MD Anderson Cancer Center, Houston, TX, USA; ^4^Department of Abdominal Imaging, The University of Texas MD Anderson Cancer Center, Houston, TX, USA; ^5^Department of Hematopathology, The University of Texas MD Anderson Cancer Center, Houston, TX, USA; ^6^Department of Pathology, The University of Texas MD Anderson Cancer Center, Houston, TX, USA; ^7^Steele Laboratories, Department of Radiation Oncology, Massachusetts General Hospital and Harvard Medical School, Boston, MA, USA; ^8^Department of Clinical Oncology, Faculty of Medicine, Ain Shams University, Cairo, Egypt

**Keywords:** IGF-1, Child-Pugh, sorafenib, liver reserve, hepatocellular carcinoma

## Abstract

Background: Sorafenib was the first systemic therapy approved for the treatment of Child-Turcotte-Pugh (CTP) class A patients with advanced hepatocellular carcinoma (HCC). However, there are no biomarkers to predict survival and treatment outcomes and guide HCC systemic therapy. Type 1 insulin-like growth factor (IGF-1)/CTP composite score has emerged as a potential hepatic reserve assessment tool. Our study investigated the association of the IGF/CTP score with overall survival (OS) and progression-free survival (PFS) of HCC patients treated with sorafenib.

Materials and Methods: In this prospective study, patients with HCC were treated with sorafenib and followed up until progression/death. We calculated the IGF/CTP score and used the Kaplan-Meier method and log-rank test to estimate and compare the time-to-event outcomes between patient subgroups.

Results: 171 patients were included, 116 of whom were CTP class A. Median PFS for IGF/CTP score AA and AB patients were 6.88 and 4.28 months, respectively (*p* = 0.1359). Median OS for IGF/CTP score AA and AB patients were 14.54 and 7.60 months, respectively (*p* = 0.1378). The PFS and OS was superior in AA patients, but the difference was not significant, likely due to the sample size. However, there was a significant difference in early OS and PFS curves between AA and AB (*p* = 0.0383 and *p* = 0.0099), respectively.

Conclusions: In CTP class A patients, IGF/CTP score B was associated with shorter PFS and OS, however, study was underpowered to reach statistical significance. If validated in larger cohorts, IGF/CTP score may serve as stratification tool in clinical trials, a hepatic reserve assessment tool for HCC outcomes prediction and to assist in therapy decisions.

## INTRODUCTION

Accurate assessment of the functional hepatic reserve is important to the prognostic and treatment prediction for patients with liver disease [1, 2]. The Child-Turcotte-Pugh (CTP) qualitative scoring system is used to assess severity of cirrhosis, survival prospects hepatic reserve, guiding treatment decisions, and stratifying patients with hepatocellular carcinoma (HCC) for clinical trial entry; It classifies patients with liver disease into 3 groups (A, B, and C), this classification is based on bilirubin and albumin levels, prothrombin time, and subjective assessments of encephalopathy and ascites [1, 2].

Several prospective and retrospective studies have confirmed that HCC patients in CTP classes B and C showed worse prognoses and accelerated decline in liver function as compared with CTP A patients [3, 4]. Accordingly, clinical trials studying systemic therapies for HCC; include only those with CTP A classification, to assess the effects of the systemic therapy under investigation without the confounding issues of hepatic failure or death which may result from an underlying poor liver reserves [3–5]. However, it is known now that the clinical outcomes for patients can vary even within those in same CTP class, including class A. In addition, subjective variables, ascites and encephalopathy of the CTP scoring system have been considered its major shortcoming because they are difficult to grade, vary daily, and could be affected by symptomatic management [6–11].

The majority of circulating insulin–like growth factor (IGF) is synthesized and secreted by the liver [12]. Therefore, circulating IGF-1 level has been shown to decrease dramatically in Chronic liver disease and HCC [13–18]; We recently reported a significant correlation between advanced clinicopathologic parameters and low IGF-1 levels HCC patients [19, 20]. Subsequently, we prospectively validated a revised CTP scoring system by replacing the subjective clinical assessment of ascites and encephalopathy in the CTP score with objectively quantified plasma IGF-1 levels to create new IGF/CTP score classes (IGF/CTP-A, -B, and -C, with A indicating low risk, B indicating intermediate risk, and C indicating high risk). This new scoring system was significantly associated with HCC survival prediction [21–23]. In addition, our results showed that a significant number of patients in the old CTP class A were reassigned as IGF/CTP-B or -C and had significantly poorer survival than did those in IGF/CTP-A, proving the usefulness of the IGF/CTP composite score to refine the CTP scoring system’s prognostic accuracy. Our previously published work incorporating IGF-1 into BCLC and CLIP scores showed higher prognostic value [24], and our recently published paper incorporated IGF-1 into MELD score, which significantly improved the score’s ability to predict OS in 2 independent cohorts of HCC patients [25]. However, we did not assess its predictive ability in patients treated with systemic therapy. Sorafenib, the first drug approved for the treatment of HCC, was approved after randomized, placebo-controlled trials demonstrated that it improved the overall survival (OS) of CTP class A patients with advanced HCC [26, 27]; the purpose of our current study was to investigate the usefulness of the IGF/CTP score in predicting overall survival (OS) and progression-free survival (PFS) in CTP class A patients with advanced HCC who were treated with sorafenib. Our overall hypothesis was that the IGF/CTP score could divide CTP A patients into more refined subgroups on the basis of OS and PFS.

## RESULTS

### Patient characteristics

A total of 171 patients from MD Anderson meeting our criteria were screened and included into our study. Baseline characteristics (Table 1); Most patients (98%) were classified under the Barcelona Clinic Liver Cancer (BCLC) stage C. Notably, 75% of the patients were 70 years or younger at the time of diagnosis, and 77.2% were males. During the follow-up period, 100 patients died. The median OS was 11.48 months (95% CI, 8.75–15.33 months; Supplementary Table 1) and the median follow-up time was 19.9 months (95% CI, 14.5–33.8 months). One hundred twenty-two patients had disease progression on sorafenib or died. The estimated median PFS was 5.30 months (95% CI, 4.70–6.84 months; Supplementary Table 1). The estimated 1-year failure-free probability was 19% (95% CI, 0.13–0.27).

**Table 1:** Baseline clinical characteristics of 171 patients with hepatocellular carcinoma treated with sorafenib

Variable	No. of patients (%)
Age, years	
≤ 40	2 (1.2)
41–50	7 (4.1)
51–60	48 (28.1)
61–70	71 (41.5)
> 70	43 (25.1)
Diabetes	
No	95 (55.6)
Yes	71 (41.5)
Unknown	5 (2.9)
Sex	
Female	39 (22.8)
Male	132 (77.2)
History of drinking alcohol	
No	85 (49.7)
Yes	75 (43.9)
Unknown	11 (6.4)
Family history of liver cancer	
No	136 (79.5)
Yes	7 (4.1)
Unknown	28 (16.4)
Personal History of non-HCC cancer	
No	136 (79.5)
Yes	28 (16.4)
Unknown	7 (4.1)
Race	
Asian	2 (1.2)
Black	24 (14.0)
Hispanic	9 (5.3)
White	129 (75.4)
Unknown	7 (4.1)
History of tobacco use	
No	56 (32.7)
Yes	104 (60.8)
Unknown	11 (6.4)
Ascites	
Moderate	21 (12.3)
None	119 (69.6)
Slight	31 (18.1)
Evidence of cirrhosis	
No	37 (21.6)
Yes	126 (73.7)
Unknown	8 (4.7)
ECOG performance status	
0	24 (14.0)
1	138 (80.7)
2	9 (5.3)
CTP class	
A	116 (67.8)
B	55 (32.2)
Metastasis	
No	99 (57.9)
Yes	64 (37.4)
Unknown	8 (4.7)
Tumor nodularity	
Multinodular	125 (73.1)
Uninodular	46 (26.9)
Vascular invasion	
No	78 (45.6)
Yes	83 (48.5)
Unknown	10 (5.8)
Lymph node metastasis	
No	93 (54.4)
Yes	70 (40.9)
Unknown	8 (4.7)
BCLC stage	
A	1 (0.6)
B	2 (1.2)
C	168 (98.2)

Abbreviations: BCLC, Barcelona Clinic Liver Cancer; CTP, Child-Turcotte-Pugh; ECOG, Eastern Cooperative Oncology Group.

### Circulating IGF-1 is associated with survival in sorafenib-treated patients

OS and PFS significantly differed between groups of patients when stratified by IGF-1 levels. Compared to patients with high IGF-1 levels (> 26.0 ng/mL), patients with low IGF-1 levels (≤ 26.0 ng/mL) had shorter median OS durations (12.83 months [95% CI, 10.62–20.62 months] vs. 5.23 months [95% CI, 4.28–10.66 months], respectively; *p* = 0.003) and shorter median PFS durations (6.05 months [95% CI, 4.97–9.41 months] vs. 4.18 months [95% CI, 2.99–5.23 months], respectively; *p* = 0.02).

### IGF/CTP score is associated with survival in sorafenib-treated patients

Both the CTP and the IGF/CTP scores classified patients into three risk groups that differed significantly in terms of OS and PFS; low (A)-, intermediate (B)-, and high (C). After IGF/CTP scoring, 87 of 116 CTP class A patients were reclassified as IGF/CTP-A (AA) and 29 patients were reclassified as IGF/CTP-B (AB) (Supplementary Table 1). Seven of 55 CTP class B patients were reclassified as IGF/CTP-A (BA), 35 were reclassified as IGF/CTP-B (BB), and 13 were reclassified as IGF/CTP-C (BC).

**Figure 1:**
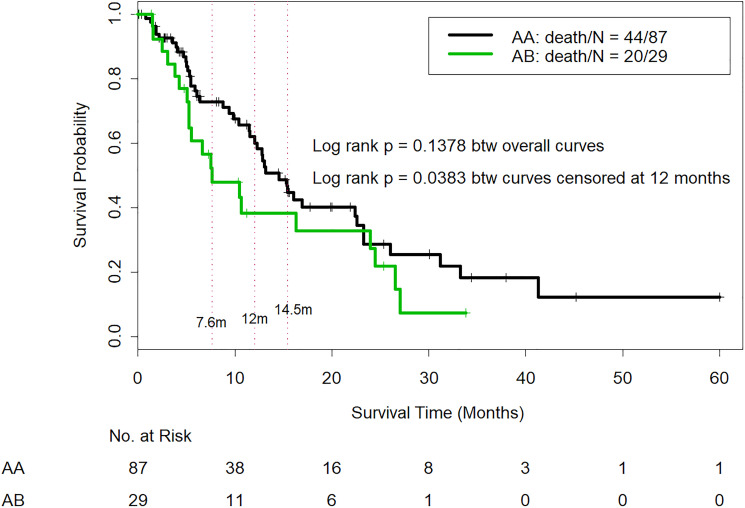
Comparison of overall survival in CTP class A patients who were reclassified as IGF/CTP-A (AA) and or IGF/CTP-B (AB). -Log-rank test was used to compare OS curves among patients who survived longer than 12.00 months; these patients were censored at 12.00 months. The log-rank test indicated that AA patients had significantly longer OS than AB patients (*p* = 0.038).

Among the patients classified as CTP class A, the median OS duration was 14.54 months for those reclassified as AA and 7.60 months for those reclassified as AB (*p* = 0.13); although the OS difference favored the AA patients, the *p value* was not significant. The hazard ratio (HR) for AA vs. AB patients was 1.49, and our study power calculation suggested that 325 participants with 220 events would be needed to achieve the 80% power needed to detect this HR, although the current data had only 39% power. We used the Wald test to compare OS probabilities between AA and AB patients at 7.60 months, 12.00 months, and 14.54 months (Table 2). The analysis indicated that AA patients had a significantly higher OS probability at 7.60 months (Wald test *p value* = 0.030) and at 12.00 months (Wald test *p value* = 0.046). At 14.54 months, the OS probability was higher in AA patients but not statistically significant (Wald test *p value* = 0.49). In addition, the log-rank test was used to compare OS curves censored at 12 months, i.e., patients who survived longer than 12.00 monthswere censored at 12.00 months. The log-rank test indicated that AA patients had significantly longer OS than AB patients (*p* = 0.038) (Figure 1).

**Table 2:** Comparison of OS probabilities for AA and AB patients at 7.60, 12.00, and 14.54 months

IGF/CTP classification	OS probability at 7.60 months (95% CI)	Wald test *p* value
AA	0.73 (0.63 , 0.84 )	0.0296
AB	0.48 (0.32 , 0.72 )	
	**OS probability at 12.00 months (95% CI)**	**Wald test *p* value**
AA	0.62 (0.51 , 0.75 )	0.046
AB	0.38 (0.23 , 0.64 )	
	**OS probability at 14.54 months (95% CI)**	**Wald test *p* value**
AA	0.49 (0.37 , 0.63 )	0.4915
AB	0.38 (0.23 , 0.64 )	

Abbreviations: CTP, Child-Turcotte-Pugh; IGF, insulin-like growth factor-1; OS, overall survival.

**Figure 2:**
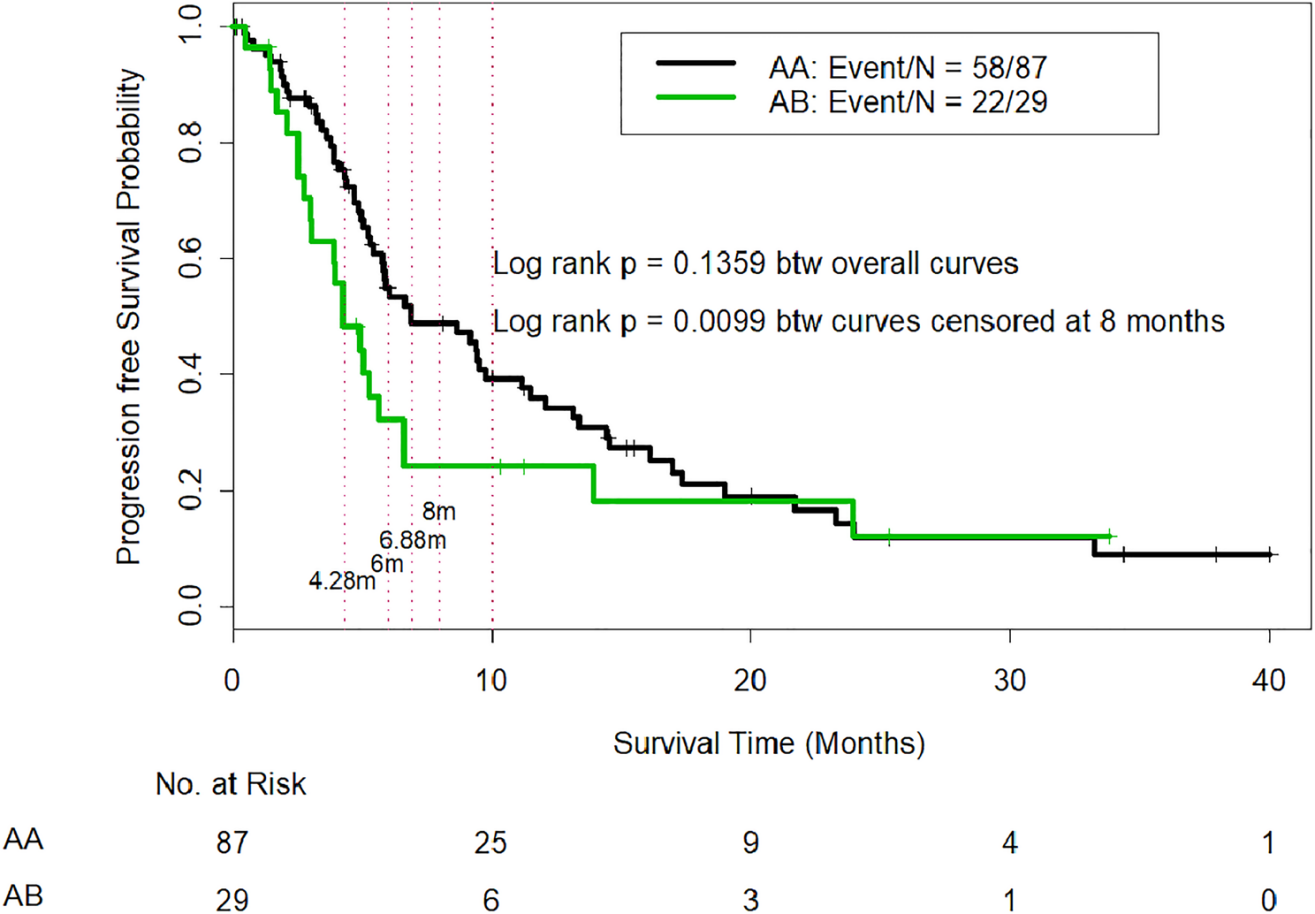
Comparison of progression-free survival in CTP class A patients who were reclassified as IGF/CTP-A (AA) and or IGF/CTP-B (AB). -Log-rank test was used to compare PFS curves among patients who survived longer than 8.00 months without disease progression; these patients were censored at 8.00 months. The log-rank test indicated that AA patients had significantly better early PFS than AB patients (*p* = 0.0099).

The median PFS time was 6.88 months for AA patients and 4.28 months for AB patients (*p* = 0.1378), and although the PFS difference favored the AA patients, the *p value* was not significant. However, our study was underpowered to detect statistically significant differences in the median PFS because of its small sample size. We used the Wald test to compare the PFS probabilities between AA and AB patients at 4.28 months, 5.00 months, 6.00 months, 6.88 months, and 8.00 months (Table 3). The analysis indicated that the AA patients had a significantly higher OS probability at 4.28 months (Wald test *p value* = 0.012), at 5.00 months (Wald test *p value* = 0.043), at 6.00 months (Wald test *p value* = 0.036), at 6.88 months (Wald test *p value* = 0.017), and at 8.00 months (Wald test *p value* = 0.017). The PFS probability was higher in the AA patients at 10.00 and 12.00 months (Wald test *p value*s = 0.14 and 0.25, respectively), but the differences were not statistically significant. In addition, the log-rank test was used to compare PFS curves censored at 8 months, i.e., patients who survived longer than 8.00 months without disease progression were censored at 8.00 months. The log-rank test indicated that AA patients had significantly better early PFS than AB patients (*p* = 0.0099) (Figure 2).

**Table 3:** Comparison of PFS probabilities for AA and AB patients at 4.28, 5.00, 6.00, 6.88, and 8.00 months

IGF/CTP classification	PFS probability at 4.28 months (95% CI)	Wald test *p* value
AA	0.75 (0.66, 0.86 )	0.0122
AB	0.48 (0.33, 0.71 )	
	**PFS probability at 5.00 months (95% CI)**	**Wald test *p* value**
AA	0.67 (0.57, 0.78 )	0.0431
AB	0.44 (0.29, 0.68 )	
	**PFS probability at 6.00 months (95% CI)**	**Wald test *p* value**
AA	0.55 (0.44, 0.68 )	0.0364
AB	0.32 (0.18, 0.56 )	
	**PFS probability at 6.88 months (95% CI)**	**Wald test *p* value**
AA	0.49 (0.38, 0.62 )	0.0171
AB	0.24 (0.12, 0.48 )	
	**PFS probability at 8.00 months (95% CI)**	**Wald test *p* value**
AA	0.49 (0.38, 0.62 )	0.0171
AB	0.24 (0.12, 0.48 )	

Abbreviations: CTP, Child-Turcotte-Pugh; IGF, insulin-like growth factor-1; PFS, progression-free survival.

OS and PFS results for the CTP class A patients by plasma IGF-1 levels are summarized in Supplementary Table 2. Patients with low IGF-1 levels (≤ 26.0 ng/mL) had worse OS and PFS than those with higher IGF-1 levels (> 26.0 ng/mL), but these differences were not statistically significant.

OS and PFS results for the CTP class B patients by plasma IGF-1 levels are summarized in Supplementary Table 3. Patients with low IGF-1 (≤ 26.0 ng/mL) had significantly worse OS and PFS than those with higher IGF-1 levels (> 26.0 ng/mL) (*p* = 0.0027 and *p* = 0.028 respectively)and they also had worse PFS.

OS and PFS results for the CTP class B patients by IGF/CTP scores (Supplementary Table 3). Of the 55 patients classified as CTP class B, 35 were reclassified as IGF/CTP-B (BB) and had a median OS of 7.07 months (95% CI, 3.98–21.58 months) and a median PFS of 4.54 months (95% CI, 3.82–10.26 months). Seven CTP class B patients were reclassified as IGF/CTP-A (BA) and had a median OS of 6.71 months (95% CI, 4.41 months-not applicable [N/A]) and a median PFS of 1.97 months (95% CI, 1.84 months-N/A). Thirteen CTP class B patients were reclassified as IGF/CTP-C (BC) and had a median OS of 4.18 (95% CI, 2.07 months-N/A) and a median PFS of 3.29 months (95% CI, 2.07 months-N/A).

To evaluate whether IGF-1 provides additional prognostic effects on OS and PFS to CTP in this dataset, Cox models were fitted to include both CTP and IGF-1. The analysis showed that IGF-1 provided additional prognostic effects (HR = 1.87 and *p* = 0.0054) for OS, (HR = 1.59 and *p* = 0.0319) for PFS. (Supplementary Table 1). To assess the performance of IGF-modified-CTP and CTP in their predictive capacity of OS and PFS, Harrell’s C-index was calculated for each of score systems, and U statistics was used to compare the c-indices between the two score systems. C-index for IGF-CTP was slightly higher than for CTP, but it was not statistically significant (*p* = 0.61 for OS and *p* = 0.96 for PFS, respectively, Table 4).

**Table 4:** Estimated C-index for CTP, IGF-modified-CTP in all patients and in CTP A patients, as well as for Cox models with and without IGF-1 in CTP A patients

All patients		C Index (95% CI)	*P* value
**OS**	CTP	0.58 (0.53, 0.63)	0.61
	IGF-CTP	0.60 (0.54, 0.66)	
**PFS**	CTP	0.56 (0.52, 0.61)	0.96
	IGF-CTP	0.57 (0.52, 0.62)	
**CTP A patients**			
**OS**	IGFCPT	0.55 ( 0.49, 0.61)	
**PFS**	IGFCPT	0.56 (0.51, 0.61)	
**OS**	Model with albumin + bilirubin + INR	0.64 (0.56, 0.73)	0.026
	Model with albumin + bilirubin + INR + IGF1	0.65 (0.57, 0.74)	
**PFS**	Model with albumin + bilirubin + INR	0.61 (0.54, 0.68)	0.11
	Model with albumin + bilirubin + INR + IGF1	0.63 (0.55, 0.70)	

C-index was calculated to assess the predictive capacity of IGF-modified-CTP for OS and PFS in CTP A patients. In addition, Cox models including albumin + bilirubin + INR with and without IGF-1 were fitted, and C-index was calculated for each model. The analysis showed that C-index for OS was statistically significant higher in the model with IGF-1 than that in the model without IGF-1 (*p* = 0.026, Table 4). The C-index for PFS was slightly higher in the model with IGF-1 than that in the model without IGF-1, but it was not statistically significant (*p* = 0.11) (Table 4).

In patients with CTP class A HCC, we compared the rate of adverse events among those reclassified as IGF/CTP-A (AA) to that of patients reclassified as IGF/CTP-B (AB) (Supplementary Table 4). There was a higher overall rate of adverse events in the AA patients than in the AB patients, especially in regard to grade III–IV adverse events, bleeding in the upper and lower gastrointestinal tract, nose bleeding, renal failure, liver failure, encephalopathy, fatigue, weight loss, anorexia, and vomiting.

## DISCUSSION

This study supports our hypothesis that the IGF/CTP score can distinguish and refine CTP class A patients. We found that patients who were reclassified as IGF/CTP-B or -C had poorer prognoses (shorter OS and PFS durations than those reclassified as IGF/CTP-A). However, for CTP class A patients, the threshold for statistical significance for the OS and PFS durations of the reclassified groups AA and AB were not met because of the limited power of the study (39%); our power calculation suggested that 325 participants with 220 events would be needed to achieve the 80% power needed to detect significant difference in survival outcome.

Assessing liver reserve in HCC is of a great value as a tool for stratification of patients in clinical trials as well as to predict HCC outcome and guide therapy decisions in routine practice [28]; Sorafenib, the first drug approved for the treatment of HCC, was approved after randomized, placebo-controlled trials demonstrated that it improved the overall survival (OS) of CTP class A patients with advanced HCC [26, 27] with high importance to select which patients will profit the most from sorafenib; especially that systemic therapy in HCC carries high cost which adds a significant burden to health care system budgets; thus particularly important to identify which HCC patients will benefit from systemic therapy as distinct from those in whom systemic therapy will pose lower survival benefit. In this context, applying a simple, noninvasive, routine laboratory test, relatively low cost marker for liver reserve assessment will be helpful.

Our group recently reported a significant correlation between advanced clinicopathologic parameters and lower plasma IGF-1 levels in patients with HCC, IGF-1 has emerged as a substitute biomarker for Liver reserve and we have showed the usefulness of adding it to the CTP score; substituting ascites and encephalopathy [19]. This led to our development and prospective validation of our revised and improved IGF/CTP scoring system, which replaced the subjective clinical assessment of ascites and encephalopathy included in the original CTP scoring system with objectively quantified plasma IGF-1 levels and created new IGF/CTP score classes (A-C). The IGF/CTP score is advantageous not only because it uses only objective laboratory variables that are routinely assessed in clinical practice, but also because it includes the plasma IGF-1 level, which is a standardized, Clinical Laboratory Improvement Amendments–certified test and therefore ready for implementation in routine practice. Our prior published data showed that the IGF/CTP scoring system significantly improved the prediction of survival in HCC patients [21]. Moreover, our previous results demonstrated that a significant number of patients with CTP class A were reclassified as IGF/CTP-B and -C and had significantly poorer survival than those who were reclassified as IGF/CTP-A. However, we had not previously assessed the outcomes of sorafenib-treated patients reclassified according to our new scoring system.

Our study is the first prospective validation of the IGF/CTP scoring system association with the outcomes among patients with HCC treated with sorafenib. Although our study was not powered to determine the predictive value of the IGF/CTP score in regard to median OS and PFS durations in CTP class A patients treated with sorafenib, our subset analyses of OS and PFS at different timepoints were statistically significant and, if independently validated, could change the standard approach to assessing hepatic reserve in patients with HCC.

Our current study included an exploratory analysis of 55 CTP class B patients with HCC. This is an important population to study, as most clinical trials of systemic therapies for HCC include only CTP class A patients. In our current study, CTP class B patients with HCC who were reclassified as IGF/CTP-A (BA) had OS and PFS durations of 6.71 months and 1.97 months, respectively, while those reclassified to IGF/CTP-B (BB) had OS and PFS durations of 7.07 months and 4.54 months, respectively. These findings did not reach the statistical threshold for significance because of the small number of CTP class B patients in the current study. However, our exploratory analysis of the CTP class B group based only on IGF-1 levels support the hypothesis that IGF-1 levels predict survival outcomes in patients with HCC. The analysis indicated that patients with IGF-1 levels greater than 26.0 ng/mL had significantly longer OS and PFS durations than patients with IGF levels less than or equal to 26.0 ng/mL (*p* = 0.0027). Nevertheless, these findings remain exploratory due to the small number of CTP class B patients in our study and the retrospective nature of the analysis. Therefore, larger studies of IGF-1 levels in this population are needed.

One of the limitations of our study is that it was a single-center study in a selected population. Second, it had insufficient power to reach statistical significance, however it showed a clear trend in better survival outcomes in AA patients as compared to AB as aforementioned. To validate the observations in the current study, a larger sample size will be required. However, we believe that there is critical need for future large randomized studies with enough power to confirm our results and validate the utility of IGF-1 as a predictive score for systemic therapy response.

In conclusion, our results demonstrate that, in CTP class A patients with advanced HCC treated with sorafenib, the IGF/CTP scoring system provides more accurate associations with survival than the CTP score. This finding should be validated in studies with larger sample sizes. If our results are validated in independent future studies, our approach of computing CTP scores using IGF-1 levels and other laboratory-based parameters that are less subjective than the clinical assessments currently used may lead to a paradigm shift in predicting the efficacy and toxicity of systemic HCC therapies and in stratifying patients in HCC clinical trials. Our approach could also help differentiate between CTP class A patients who may benefit from active therapy and those in whom active therapy should be deferred to avoid unnecessary harm and save health care resources.

## MATERIALS AND METHODS

### Study design and study population

The University of Texas MD Anderson Cancer Center’s Institutional Review Board approved this prospective study, and informed consent was obtained from all enrolled patients. Our study was prospectively designed to collect pretreatment samples from sorafenib patients, follow them until progression, and analyze correlation with pretreatment IGF-1 score. Therefore, score analysis was done after therapy was concluded for all patients. Adult patients with pathologically or radiologically confirmed HCC, as defined by the American Association for the Study of Liver Diseases, who were treated at MD Anderson Cancer Center from January 2004 to July 2018 were enrolled in the study. Patients’ blood samples and epidemiologic and clinical data were collected, and plasma samples were analyzed retrospectively for IGF-1. Peripheral venous blood specimens (3–5 mL) were collected from all patients; anticoagulated with ethylenediaminetetraacetic acid, and centrifuged for 15 minutes at 3000 rpm. The plasma was then removed, aliquoted, and snap frozen at –20°C. Baseline IGF-1 levels, clinical characteristics, Adverse events to treatment using CTCAE V4.03; and epidemiological data were retrieved from the medical records. IGF/CTP scores were calculated and patients were classified as IGF/CTP-A, -B, or -C based on their serum bilirubin and albumin levels, prothrombin times, and plasma IGF-1 levels (≤ 26 ng/ml: score of 3, > 26 and ≤ 50, score of 2 and score of 1 for IGF > 50); IGF-CTP scores have a range between 4–12; 4–5 for IGF-CTP(A), 6–7 for IGF-CTP (B) and > 7 for IGF-CTP (C) (Table 5); PFS was calculated from the date that sorafenib treatment began to the date of disease progression or death. OS was calculated from the date that sorafenib treatment began to the date of death or of the last follow-up.

**Table 5:** IGF/CTP scoring system

	IGF/CTP score (points)^†^		
Variable	1	2	3
Encephalopathy	None	Mild (1–2)	Severe (3–4)
Ascites	None	Mild/moderate	Severe/refractory
Albumin, g/dL	> 3.5	2.8–3.5	< 2.8
PT, s	< 4	4–6	> 6
Bilirubin, mg/dL^‡^	< 2	2–3	> 3
IGF-1, ng/mL	> 50	26–50	< 26

^†^IGF/CTP score classes: A, 4–5 points; B, 6–7 points; C, more than 7 points. ^‡^In patients with primary biliary cirrhosis and primary sclerosing cholangitis, the upper limit of bilirubin for 1 point is 4 mg/dL and the upper limit for 2 points is 10 mg/dL. Abbreviations: CTP, Child-Turcotte-Pugh; IGF-1, type 1 insulin-like growth factor; PT, prothrombin time.

Frequencies and percentages were used to tabulate patients’ baseline characteristics and adverse events. The Fisher exact test was used to compare the occurrence of adverse events between subgroups of patients. The Kaplan-Meier method was used to estimate time-to-event outcomes (e.g., OS, PFS), and the log-rank test and Cox proportional hazards models was used to compare the time-to-event outcomes. In addition, we used the Wald test to compare OS and PFS probabilities between AA and AB patients at some early survival time points; Harrell’s concordance index (c-index) and U statistics [29] were used to evaluate the predictive capacity between CTP and IGF-CTP score systems and between Cox models with and without IGF-1. A *p value* if less than 0.05 was considered statistically significant. S+ version 8.2.0 (TIBCO Software Inc., Palo Alto, CA, USA) and SAS version 9.4 (SAS Institute, Cary, NC, USA) were used for the analyses.

## SUPPLEMENTARY MATERIALS











## Abbreviations

CTP: Child-Turcotte-Pugh
HCC: hepatocellular carcinoma
HR: hazard ratio
IGF-1: type 1 insulin-like growth factor
N/A: not applicable
OS: overall survival
PFS: progression-free survival

## References

[1] Vauthey JN , Dixon E , Abdalla EK , Helton WS , Pawlik TM , Taouli B , Brouquet A , Adams RB , and American Hepato-Pancreato-Biliary Association, and Society of Surgical Oncology, and Society for Surgery of the Alimentary Tract. Pretreatment assessment of hepatocellular carcinoma: expert consensus statement. HPB (Oxford). 2010; 12:289–99. 10.1111/j.1477-2574.2010.00181.x. 20590901 PMC2951814

[2] Pugh RN , Murray-Lyon IM , Dawson JL , Pietroni MC , Williams R . Transection of the oesophagus for bleeding oesophageal varices. Br J Surg. 1973; 60:646–9. 10.1002/bjs.1800600817. 4541913

[3] Llovet JM , Di Bisceglie AM , Bruix J , Kramer BS , Lencioni R , Zhu AX , Sherman M , Schwartz M , Lotze M , Talwalkar J , Gores GJ , and Panel of Experts in HCC-Design Clinical Trials. Design and endpoints of clinical trials in hepatocellular carcinoma. J Natl Cancer Inst. 2008; 100:698–711. 10.1093/jnci/djn134. 18477802

[4] Wilson SR , Greig P , Kaseb AO . Pretreatment assessment of hepatocellular cancer: expert consensus conference. HPB (Oxford). 2010; 12:300–01. 10.1111/j.1477-2574.2010.00187.x. 20590902 PMC2951815

[5] Morris JS , Hassan MM , Zohner YE , Wang Z , Xiao L , Rashid A , Haque A , Abdel-Wahab R , Mohamed YI , Ballard KL , Wolff RA , George B , Li L , et al. HepatoScore-14: measures of biological heterogeneity significantly improve prediction of hepatocellular carcinoma risk. Hepatology. 2020 9 15. 10.1002/hep.31555. [Epub ahead of print]. 32931023 PMC7956911

[6] Durand F , Valla D . Assessment of the prognosis of cirrhosis: Child-Pugh versus MELD. J Hepatol. 2005 (Suppl); 42:S100–07. 10.1016/j.jhep.2004.11.015. 15777564

[7] Kim HJ , Lee HW . Important predictor of mortality in patients with end-stage liver disease. Clin Mol Hepatol. 2013; 19:105–15. 10.3350/cmh.2013.19.2.105. 23837134 PMC3701842

[8] Oellerich M , Burdelski M , Lautz HU , Rodeck B , Duewel J , Schulz M , Schmidt FW , Brodehl J , Pichlmayr R . Assessment of pretransplant prognosis in patients with cirrhosis. Transplantation. 1991; 51:801–06. 10.1097/00007890-199104000-00013. 2014533

[9] Pessione F , Ramond MJ , Peters L , Pham BN , Batel P , Rueff B , Valla DC . Five-year survival predictive factors in patients with excessive alcohol intake and cirrhosis. Effect of alcoholic hepatitis, smoking and abstinence. Liver Int. 2003; 23:45–53. 10.1034/j.1600-0676.2003.01804.x. 12640727

[10] Said A , Williams J , Holden J , Remington P , Gangnon R , Musat A , Lucey MR . Model for end stage liver disease score predicts mortality across a broad spectrum of liver disease. J Hepatol. 2004; 40:897–903. 10.1016/j.jhep.2004.02.010. 15158328

[11] Testa R , Valente U , Risso D , Caglieris S , Giannini E , Fasoli A , Botta F , Dardano G , Lantieri PB , Celle G . Can the MEGX test and serum bile acids improve the prognostic ability of Child-Pugh’s score in liver cirrhosis? Eur J Gastroenterol Hepatol. 1999; 11:559–63. 10.1097/00042737-199905000-00016. 10755262

[12] Colakoğlu O , Taşkiran B , Colakoğlu G , Kizildağ S , Ari Ozcan F , Unsal B . Serum insulin like growth factor-1 (IGF-1) and insulin like growth factor binding protein-3 (IGFBP-3) levels in liver cirrhosis. Turk J Gastroenterol. 2007; 18:245–49. 18080921

[13] Buzzelli G , Dattolo P , Pinzani M , Brocchi A , Romano S , Gentilini P . Circulating growth hormone and insulin-like growth factor-I in nonalcoholic liver cirrhosis with or without superimposed hepatocarcinoma: evidence of an altered circadian rhythm. Am J Gastroenterol. 1993; 88:1744–48. 8213718

[14] Luo SM , Tan WM , Deng WX , Zhuang SM , Luo JW . Expression of albumin, IGF-1, IGFBP-3 in tumor tissues and adjacent non-tumor tissues of hepatocellular carcinoma patients with cirrhosis. World J Gastroenterol. 2005; 11:4272–76. 10.3748/wjg.v11.i27.4272. 16015705 PMC4615458

[15] Stuver SO , Kuper H , Tzonou A , Lagiou P , Spanos E , Hsieh CC , Mantzoros C , Trichopoulos D . Insulin-like growth factor 1 in hepatocellular carcinoma and metastatic liver cancer in men. Int J Cancer. 2000; 87:118–21. 10.1002/1097-0215(20000701)87:1<118::AID-IJC17>3.0.CO;2-W. 10861461

[16] Su WW , Lee KT , Yeh YT , Soon MS , Wang CL , Yu ML , Wang SN . Association of circulating insulin-like growth factor 1 with hepatocellular carcinoma: one cross-sectional correlation study. J Clin Lab Anal. 2010; 24:195–200. 10.1002/jcla.20320. 20486202 PMC6647566

[17] García-Galiano D , Sánchez-Garrido MA , Espejo I , Montero JL , Costán G , Marchal T , Membrives A , Gallardo-Valverde JM , Muñoz-Castañeda JR , Arévalo E , De la Mata M , Muntané J . IL-6 and IGF-1 are independent prognostic factors of liver steatosis and non-alcoholic steatohepatitis in morbidly obese patients. Obes Surg. 2007; 17:493–503. 10.1007/s11695-007-9087-1. 17608262

[18] Völzke H , Nauck M , Rettig R , Dörr M , Higham C , Brabant G , Wallaschofski H . Association between hepatic steatosis and serum IGF1 and IGFBP-3 levels in a population-based sample. Eur J Endocrinol. 2009; 161:705–13. 10.1530/EJE-09-0374. 19690083

[19] Kaseb AO , Morris JS , Hassan MM , Siddiqui AM , Lin E , Xiao L , Abdalla EK , Vauthey JN , Aloia TA , Krishnan S , Abbruzzese JL . Clinical and prognostic implications of plasma insulin-like growth factor-1 and vascular endothelial growth factor in patients with hepatocellular carcinoma. J Clin Oncol. 2011; 29:3892–99. 10.1200/JCO.2011.36.0636. 21911725 PMC3189091

[20] Kaseb AO , Abbruzzese JL , Vauthey JN , Aloia TA , Abdalla EK , Hassan MM , Lin E , Xiao L , El-Deeb AS , Rashid A , Morris JS . I-CLIP: improved stratification of advanced hepatocellular carcinoma patients by integrating plasma IGF-1 into CLIP score. Oncology. 2011; 80:373–81. 10.1159/000329040. 21822028 PMC3171278

[21] Kaseb AO , Xiao L , Hassan MM , Chae YK , Lee JS , Vauthey JN , Krishnan S , Cheung S , Hassabo HM , Aloia T , Conrad C , Curley SA , Vierling JM , et al. Development and validation of insulin-like growth factor-1 score to assess hepatic reserve in hepatocellular carcinoma. J Natl Cancer Inst. 2014; 106:dju088. 10.1093/jnci/dju088. 24815863 PMC4085880

[22] Lacin S , Yalcin S , Karakas Y , Hassan MM , Amin H , Mohamed YI , Rashid A , Morris JS , Xiao L , Qayyum A , Kaseb AO . Prognostic Significance of Serum Insulin-Like Growth Factor-1 in Hepatocellular Cancer Patients: A Validation Study. J Hepatocell Carcinoma. 2020; 7:143–53. 10.2147/JHC.S258930. 32984091 PMC7502406

[23] Abdel-Wahab R , Shehata S , Hassan MM , Xiao L , Lee JS , Cheung S , Essa HH , Hassabo HM , Shalaby AS , Mosad E , Raghav K , Rashid A , Wolff RA , et al. Validation of an IGF-CTP scoring system for assessing hepatic reserve in Egyptian patients with hepatocellular carcinoma. Oncotarget. 2015; 6:21193–207. 10.18632/oncotarget.4176. 26098859 PMC4673259

[24] Abdel-Wahab R , Lacin S , Shalaby AS , Hassan M , Xiao L , Morris J , Amin HM , Kaseb AO . Integrating insulin-like growth factor-1 (IGF-1) score into Barcelona Clinic Liver Cancer (BCLC) and Cancer of the Liver Italian Program (CLIP) scores. J Clin Oncol. 2016; 34:4081. 10.1200/JCO.2016.34.15_suppl.4081.

[25] Abdel-Wahab R , Hassan MM , George B , Carmagnani Pestana R , Xiao L , Lacin S , Yalcin S , Shalaby AS , Al-Shamsi HO , Raghav K , Wolff RA , Yao JC , Girard L , et al. Impact of Integrating Insulin-Like Growth Factor 1 Levels into Model for End-Stage Liver Disease Score for Survival Prediction in Hepatocellular Carcinoma Patients. Oncology. 2020; 98:836–46. 10.1159/000502482. 33027788 PMC7704605

[26] Cheng AL , Kang YK , Chen Z , Tsao CJ , Qin S , Kim JS , Luo R , Feng J , Ye S , Yang TS , Xu J , Sun Y , Liang H , et al. Efficacy and safety of sorafenib in patients in the Asia-Pacific region with advanced hepatocellular carcinoma: a phase III randomised, double-blind, placebo-controlled trial. Lancet Oncol. 2009; 10:25–34. 10.1016/S1470-2045(08)70285-7. 19095497

[27] Llovet JM , Ricci S , Mazzaferro V , Hilgard P , Gane E , Blanc JF , de Oliveira AC , Santoro A , Raoul JL , Forner A , Schwartz M , Porta C , Zeuzem S , et al, and SHARP Investigators Study Group. Sorafenib in advanced hepatocellular carcinoma. N Engl J Med. 2008; 359:378–90. 10.1056/NEJMoa0708857. 18650514

[28] El-Serag HB , Rudolph KL . Hepatocellular carcinoma: epidemiology and molecular carcinogenesis. Gastroenterology. 2007; 132:2557–76. 10.1053/j.gastro.2007.04.061. 17570226

[29] Harrell FE Jr , Lee KL , Mark DB . Multivariable prognostic models: issues in developing models, evaluating assumptions and adequacy, and measuring and reducing errors. Stat Med. 1996; 15:361–87. 10.1002/(sici)1097-0258(19960229)15:4<361::Aid-sim168>3.0.Co;2-4. 8668867

