# Supplementary material for: Insulin-like growth factor 1/Child-Turcotte-Pugh composite score as a predictor of treatment outcomes in patients with advanced hepatocellular carcinoma treated with sorafenib
Source: Oncotarget. 2021 Apr 13;12(8):756–66. doi: 10.18632/oncotarget.27924 (PMC8057275; doi:10.18632/oncotarget.27924)
Supplement: Supplementary file 1 [file oncotarget-12-756-s001.pdf]

Insulin-like growth factor 1/Child-Turcotte-Pugh composite score as a predictor of treatment outcomes in patients with advanced hepatocellular carcinoma treated with sorafenib

SUPPLEMENTARY MATERIALS

Supplementary Table 1: Log-rank test and Cox model results for OS and PFS among patient subgroups. See Supplementary Table 1

Supplementary Table 2: Log-rank test comparing OS and PFS among CTP class A patient subgroups. See Supplementary Table 2

Supplementary Table 3: Log-rank test comparing OS and PFS among CTP class B patient subgroups. See Supplementary Table 3

Supplementary Table 4: Adverse events in HCC patients with CTP class A. See Supplementary Table 4
