# Supplementary material for: Insulin-like growth factor 1/Child-Turcotte-Pugh composite score as a predictor of treatment outcomes in patients with advanced hepatocellular carcinoma treated with sorafenib
Source: Oncotarget. 2021 Apr 13;12(8):756–66. doi: 10.18632/oncotarget.27924 (PMC8057275; doi:10.18632/oncotarget.27924)
Supplement: Supplementary file 2 [file oncotarget-12-756-s002.docx]

**Supplementary Table 1: Log-rank test *and* Cox model results for OS and PFS among patient subgroups**

|  | **N** | **E** | **Median OS (95% CI)** | **OS rate at 1 year (95% CI)** | ***P* value** | **E*** | **Median PFS (95% CI)** | **PFS rate at 1 year (95% CI)** | ***P* value** |
| --- | --- | --- | --- | --- | --- | --- | --- | --- | --- |
| All patients | 171 | 100 | 11.48 (8.75 , 15.33) | 0.48 (0.4 , 0.57) |  | 122 | 5.30 (4.70 , 6.84) | 0.27 (0.2 , 0.36) |  |
| **IGF-1 level** |  |  |  |  |  |  |  |  |  |
| >26 | 134 | 72 | 12.83 (10.62 , 20.62) | 0.55 ( 0.45 , 0.66 ) | 0.0039 | 93 | 6.05 (4.97 , 9.41) | 0.31 (0.23 , 0.41) | 0.0213 |
| ≤26 | 37 | 28 | 5.23 (4.28 , 10.66) | 0.25 ( 0.13 , 0.47 ) |  | 29 | 4.18 (2.99 , 5.23) | 0.14 (0.06 , 0.34) |  |
| **IGF-1a level** |  |  |  |  |  |  |  |  |  |
| >50 | 79 | 41 | 13.16 (11.48 , 22.4) | 0.6 ( 0.48 , 0.75 ) | 0.0072 | 54 | 6.64 (5.43 , 11.15) | 0.32 (0.22 , 0.47) | 0.0478 |
| 26-50 | 55 | 31 | 11.78 (5.07 , 23.95) | 0.47 (0.34, 0.65) |  | 39 | 4.93 (3.91 , 10.26) | 0.28 (0.17 , 0.46) |  |
| ≤26 | 37 | 28 | 5.23 (4.28 , 10.66) | 0.25 (0.13 , 0.47) |  | 29 | 4.18 (2.99 , 5.23) | 0.14 (0.06 , 0.34) |  |
| **CTP score** |  |  |  |  |  |  |  |  |  |
| A | 116 | 64 | 12.83 (10.66 , 22.4) | 0.56 (0.46 , 0.67) | 0.002 | 80 | 5.89 (5.03 , 9.41) | 0.33 (0.25 , 0.44) | 0.0075 |
| B | 55 | 36 | 7.07 (4.41 , 11.78) | 0.29 (0.17 , 0.49) |  | 42 | 4.05 (2.96 , 7.63) | 0.14 (0.06 , 0.3) |  |
| **IGF/CTP score** |  |  |  |  |  |  |  |  |  |
| A | 94 | 49 | 13.16 (11.51 , 22.4) | 0.59 (0.49 , 0.72) | 0.0009 | 65 | 6.84 (5.3 , 10.89) | 0.33 (0.24 , 0.46) | 0.0576 |
| B | 64 | 42 | 7.5 (5.23 , 19.97) | 0.37 (0.26 , 0.54) |  | 48 | 4.41 (3.91 , 6.58) | 0.21 (0.12 , 0.36) |  |
| C | 13 | 9 | 4.18 (2.07 , N/A) | NA |  | 9 | 3.29 (2.07 , N/A) | NA |  |
| **CTP class reclassified to IGF/CTP** |  |  |  |  |  |  |  |  |  |
| Original A to new A (AA) | 87 | 44 | 14.54 (12.04 , 23.26) | 0.62 (0.51 , 0.75) | 0.0018 | 58 | 6.88 (5.76 , 11.51) | 0.36 (0.26 , 0.5) | 0.0101 |
| Original A to new B (AB) | 29 | 20 | 7.60 (5.23 , 24.47) | 0.38 (0.23 , 0.64) |  | 22 | 4.28 (3.06 , 6.61) | 0.24 (0.12 , 0.48) |  |
| Original B to new A (BA) | 7 | 5 | 6.71 (4.41 , N/A) | 0.23 (0.04 , 1) |  | 7 | 1.97 (1.84 , N/A) | NA |  |
| Original B to new B (BB) | 35 | 22 | 7.07 (3.98 , 21.58) | 0.37 (0.21 , 0.63) |  | 26 | 4.54 (3.82 , 10.26) | 0.17 (0.07 , 0.42) |  |
| Original B to new C (BC) | 13 | 9 | 4.18 (2.07 , N/A) | NA |  | 9 | 3.29 (2.07 , N/A) | NA |  |
|  |  |  |  | **HR (95% CI)** | ***P* value** |  |  | **HR (95% CI)** | ***P* value** |
| CTP B vs. CTP A |  |  |  | 1.89 (1.25, 2.86) | 0.0028 |  |  | 1.63 (1.12, 2.39) | 0.0113 |
| IGF-1 level <=26 vs. >26 |  |  |  | 1.87 (1.20, 2091) | 0.0054 |  |  | 1.59 (1.04, 2.43) | 0.0319 |
| Original A to new B (AB) vs. Original A to new A (AA) |  |  |  | 1.49 (0.876,2.548) | 0.1403 |  |  | 1.452 (0.887,2.378) | 1.382 |

Abbreviations: N, number; E, event(death); E*, event(PD or death), CTP, Child-Turcotte-Pugh; IGF, insulin-like growth factor-1; N/A, not applicable; OS, overall survival; PFS, progression-free survival; CI, confidence interval.
