# Supplementary material for: Insulin-like growth factor 1/Child-Turcotte-Pugh composite score as a predictor of treatment outcomes in patients with advanced hepatocellular carcinoma treated with sorafenib
Source: Oncotarget. 2021 Apr 13;12(8):756–66. doi: 10.18632/oncotarget.27924 (PMC8057275; doi:10.18632/oncotarget.27924)
Supplement: Supplementary file 3 [file oncotarget-12-756-s003.docx]

**Supplementary Table 2: Log-rank test comparing OS and PFS among CTP class A patient subgroups.**

|  | **N** | **E** | **Median OS (95% CI)** | **Rate at 1 year (95% CI)** | ***P* value** | **E*** | **Median PFS (95% CI)** | **PFS rate at 1 year (95% CI)** | ***P* value** |
| --- | --- | --- | --- | --- | --- | --- | --- | --- | --- |
| **CTP class A patients** | 116 | 64 | 12.83 (10.66 , 22.4) | 0.56 (0.46 , 0.67) |  | 80 | 5.89 ( 5.03 , 9.41 ) | 0.33 (0.25 , 0.44) |  |
| **IGF-1 level** |  |  |  |  |  |  |  |  |  |
| > 26 | 97 | 50 | 13.16 (11.51 , 23.26) | 0.6 (0.5 , 0.73) | 0.2859 | 65 | 6.64 ( 5.3 , 11.15 ) | 0.35 (0.26 , 0.48) | 0.3456 |
| ≤ 26 | 19 | 14 | 7.5 (5.23 , N/A) | 0.36 (0.19 , 0.69) |  | 15 | 4.97 ( 3.06 , 13.91 ) | 0.22 (0.09 , 0.53) |  |
| **IGF-1a level** |  |  |  |  |  |  |  |  |  |
| > 50 | 66 | 35 | 14.54 (11.51 , 23.26) | 0.62 (0.5 , 0.78) | 0.3136 | 46 | 6.84 ( 5.76 , 12.04 ) | 0.35 (0.24 , 0.5) | 0.5118 |
| 26-50 | 31 | 15 | 12.83 (5.03 , N/A) | 0.55 (0.38 , 0.79) |  | 19 | 5.03 ( 3.91 , N/A ) | 0.37 (0.22 , 0.63) |  |
| ≤ 26 | 19 | 14 | 7.5 (5.23 , N/A) | 0.36 (0.19 , 0.69) |  | 15 | 4.97 ( 3.06 , 13.91 ) | 0.22 (0.09 , 0.53) |  |
| **IGF-modified-CTP score** |  |  |  |  |  |  |  |  |  |
| 4 | 57 | 29 | 13.16 (11.48 , 26.05) | 0.62 (0.49 , 0.8) | 0.0005 | 39 | 6.64 ( 5.76 , 14.54 ) | 0.39 (0.27 , 0.56) | 0.0221 |
| 5 | 30 | 15 | 15.33 (5.43 , N/A) | 0.6 (0.43 , 0.83) |  | 19 | 8.62 ( 4.31 , 14.41 ) | 0.3 (0.16 , 0.57) |  |
| 6 | 20 | 12 | 16.32 (7.6 , N/A) | 0.58 (0.38 , 0.88) |  | 14 | 5.03 ( 3.91 , N/A ) | 0.35 (0.19 , 0.66) |  |
| 7 | 9 | 8 | 5.23 (4.28 , N/A) | NA |  | 8 | 3.67 ( 2.99 , N/A ) | NA |  |
| **IGFCTP** |  |  |  |  |  |  |  |  |  |
| A (AA) | 87 | 44 | 14.54 (12.04 , 23.26) | 0.62 (0.51 , 0.75) | 0.1378 | 58 | 6.88 ( 5.76 , 11.51 ) | 0.36 (0.26 , 0.5) | 0.1359 |
| B (AB) | 29 | 20 | 7.6 (5.23 , 24.47) | 0.38 (0.23 , 0.64) |  | 22 | 4.28 ( 3.06 , 6.61 ) | 0.24 (0.12 , 0.48) |  |
| **Sex** |  |  |  |  |  |  |  |  |  |
| Female | 26 | 13 | 12.83 (8.75 , N/A) | 0.56 (0.37 , 0.85) | 0.4788 | 18 | 5.43 ( 4.7 , 17.34 ) | 0.35 (0.19 , 0.63) | 0.8366 |
| Male | 90 | 51 | 13.06 (10.66 , 23.26) | 0.55 (0.45 , 0.68) |  | 62 | 5.89 ( 5.03 , 9.41 ) | 0.32 (0.23 , 0.45) |  |
| **Ascites** |  |  |  |  |  |  |  |  |  |
| None | 107 | 62 | 12.83 (10.66 , 22.4) | 0.55 (0.46 , 0.67) | 0.5759 | 76 | 5.89 ( 5.2 , 9.41 ) | 0.33 (0.24 , 0.44) | 0.4677 |
| Slight | 9 | 2 | N/A (5.03 , N/A) | 0.62 (0.32 , 1) |  | 4 | 5.03 ( 4.31 , N/A ) | 0.42 (0.15 , 1) |  |
| **Evidence of cirrhosis** |  |  |  |  |  |  |  |  |  |
| No | 28 | 15 | 22.4 (11.48 , N/A) | 0.64 (0.47 , 0.88) | 0.3118 | 17 | 8.62 ( 4.9 , 33.26 ) | 0.41 (0.24 , 0.69) | 0.2476 |
| Yes | 84 | 49 | 12.3 (9.84 , 16.32) | 0.53 (0.42 , 0.66) |  | 61 | 5.86 ( 4.97 , 9.41 ) | 0.31 (0.22 , 0.45) |  |
| **Major Hepatic vein Portal vein Invasion** |  |  |  |  |  |  |  |  |  |
| No | 56 | 29 | 16.94 (12.76 , 26.05) | 0.65 (0.51 , 0.82) | 0.024 | 36 | 6.84 ( 5.62 , 16.94 ) | 0.42 (0.29 , 0.59) | 0.0846 |
| Yes | 50 | 33 | 9.84 (5.43 , 14.54) | 0.46 (0.33 , 0.64) |  | 41 | 5.03 ( 4.31 , 9.38 ) | 0.23 (0.13 , 0.39) |  |
| **Vascular involvement** |  |  |  |  |  |  |  |  |  |
| No | 56 | 28 | 22.4 (15.33 , 26.05) | 0.68 (0.54 , 0.84) | 0.0087 | 36 | 6.88 ( 5.76 , 17.34 ) | 0.44 (0.31 , 0.61) | 0.0507 |
| Yes | 51 | 34 | 9.84 (5.43 , 13.16) | 0.45 (0.33 , 0.63) |  | 42 | 5.03 ( 4.31 , 9.38 ) | 0.22 (0.13 , 0.38) |  |
| **Portal vein thrombosis** |  |  |  |  |  |  |  |  |  |
| No | 56 | 28 | 22.4 (15.33 , 26.05) | 0.68 (0.54 , 0.84) | 0.0087 | 36 | 6.88 ( 5.76 , 17.34 ) | 0.44 (0.31 , 0.61) | 0.0507 |
| Yes | 51 | 34 | 9.84 (5.43 , 13.16) | 0.45 (0.33 , 0.63) |  | 42 | 5.03 ( 4.31 , 9.38 ) | 0.22 (0.13 , 0.38) |  |
| **Tumor nodularity** |  |  |  |  |  |  |  |  |  |
| Multinodular | 84 | 45 | 12.3 (8.75 , 22.6) | 0.53 (0.42 , 0.67) | 0.771 | 58 | 5.3 ( 4.7 , 6.88 ) | 0.33 (0.24 , 0.46) | 0.4806 |
| Uninodular | 32 | 19 | 16.09 (11.51 , 27.04) | 0.64 (0.47 , 0.86) |  | 22 | 9.38 ( 6.05 , 16.09 ) | 0.34 (0.2 , 0.6) |  |
| **Metastasis** |  |  |  |  |  |  |  |  |  |
| No | 64 | 37 | 15.33 (12.3 , 26.51) | 0.63 (0.52 , 0.78) | 0.2413 | 2 | 6.05 ( 1.28 , N/A ) | 0.38 (0.08 , 1) | 0.033 |
| Yes | 44 | 25 | 11.48 (7.5 , 16.94) | 0.43 (0.28 , 0.66) |  | 43 | 9.14 ( 5.76 , 14.54 ) | 0.4 (0.29 , 0.56) |  |
| Unknown | 8 | 2 | 6.05 (1.28 , N/A) | 0.38 (0.08 , 1 ) |  | 35 | 4.67 ( 3.91 , 6.88 ) | 0.22 (0.11 , 0.41) |  |
| **Lymph node metastasis** |  |  |  |  |  |  |  |  |  |
| No | 63 | 37 | 12.83 (10.66 , 23.26) | 0.58 (0.46 , 0.74) | 0.8002 | 42 | 6.64 ( 5.03 , 13.36 ) | 0.38 (0.27 , 0.54) | 0.4584 |
| Yes | 45 | 25 | 13.06 (8.75 , 26.51) | 0.52 (0.37 , 0.73) |  | 36 | 5.76 ( 4.34 , 11.15 ) | 0.26 (0.15 , 0.45) |  |
| Unknown | 8 | 2 | 6.05 (1.28 , N/A) | 0.38 (0.08 , 1) |  | 2 | 6.05 ( 1.28 , N/A ) | 0.38 (0.08 , 1) |  |

Abbreviations: N, number; E, event(death); E*, event(PD or death); CTP, Child-Turcotte-Pugh; IGF, insulin-like growth factor-1; N/A, not applicable; OS, overall survival; PFS, progression-free survival.
