# Supplementary material for: Insulin-like growth factor 1/Child-Turcotte-Pugh composite score as a predictor of treatment outcomes in patients with advanced hepatocellular carcinoma treated with sorafenib
Source: Oncotarget. 2021 Apr 13;12(8):756–66. doi: 10.18632/oncotarget.27924 (PMC8057275; doi:10.18632/oncotarget.27924)
Supplement: Supplementary file 4 [file oncotarget-12-756-s004.docx]

**Supplementary Table 3: Log-rank test comparing OS and PFS among CTP class B patient subgroups**

|  | **N** | **E** | **Median OS (95% CI)** | **OS rate at 1 year (95% CI)** | ***P* value** | **E*** | **Median PFS (95% CI)** | **PFS rate at 1 year (95% CI)** | ***P* value** |
| --- | --- | --- | --- | --- | --- | --- | --- | --- | --- |
| **CTP class B patients** | 55 | 36 | 7.07 (4.41 , 11.78) | 0.29 (0.17 , 0.49) |  | 42 | 4.05 (2.96 , 7.63) | 0.14 (0.06 , 0.3) |  |
| **IGF-1 level** |  |  |  |  |  |  |  |  |  |
| > 26 | 37 | 22 | 9.77 (6.71 , 21.58) | 0.38 (0.22 , 0.64) | 0.0027 | 28 | 4.54 (3.32 , 10.26) | 0.19 (0.09 , 0.41) | 0.0275 |
| ≤ 26 | 18 | 14 | 4.18 (2.07 , N/A) | 0.1 (0.02 , 0.6) |  | 14 | 3.29 (2.07 , N/A) | NA |  |
| **IGF-1a level** |  |  |  |  |  |  |  |  |  |
| > 50 | 13 | 6 | 7.76 (7.07 , N/A) | 0.38 (0.13 , 1) | 0.0097 | 8 | 4.41 (2.57 , N/A) | 0.16 (0.03 , 0.91) | 0.0724 |
| 26-50 | 24 | 16 | 10.62 (4.41 , N/A) | 0.37 (0.2 , 0.69) |  | 20 | 4.93 (3.32 , 11.61) | 0.2 (0.09 , 0.48) |  |
| ≤ 26 | 18 | 14 | 4.18 (2.07 , N/A) | 0.1 (0.02 , 0.6) |  | 14 | 3.29 (2.07 , N/A) | NA |  |
| **IGF-modified-CTP score** |  |  |  |  |  |  |  |  |  |
| 4 | 3 | 2 | 19.57 (1.91 , N/A) | 0.67 (0.3 , 1) | 0.1682 | 3 | 1.91 (1.84 , N/A) | NA | 0.0636 |
| 5 | 4 | 3 | 6.71 (4.41 , N/A) | NA |  | 4 | 2.27 (1.51 , N/A) | NA |  |
| 6 | 19 | 10 | 20.62 (7.07 , N/A) | 0.56 (0.33 , 0.96) |  | 12 | 10.26 (7.07 , N/A) | 0.36 (0.17 , 0.77) |  |
| 7 | 16 | 12 | 4.54 (2.47 , N/A) | 0.17 (0.05 , 0.59) |  | 14 | 3.98 (2.07 , 5.2) | NA |  |
| 8 | 9 | 6 | 4.77 (2.11 , N/A) | NA |  | 6 | 3.29 (2.11 , N/A) | NA |  |
| 9 | 3 | 2 | 1.55 (1.35 , N/A) | NA |  | 2 | 1.55 (1.35 , N/A) | NA |  |
| 10 | 1 | 1 | 4.18 (N/A , N/A) | NA |  | 1 | 4.18 (N/A , N/A) | NA |  |
| **IGF/CTP classification** |  |  |  |  |  |  |  |  |  |
| BA | 7 | 5 | 6.71 (4.41 , N/A) | 0.23 (0.04 , 1) | 0.21 | 7 | 1.97 (1.84 , N/A) | NA | 0.223 |
| BB | 35 | 22 | 7.07 (3.98 , 21.58) | 0.37 (0.21 , 0.63) |  | 26 | 4.54 (3.82 , 10.26) | 0.17 (0.07 , 0.42) |  |
| BC | 13 | 9 | 4.18 (2.07 , N/A) |  |  | 9 | 3.29 (2.07 , N/A) | NA |  |
| **Sex** |  |  |  |  |  |  |  |  |  |
| Female | 12 | 9 | 3.82 (2.47 , N/A) | 0.11 (0.02 , 0.68) | 0.0885 | 10 | 3.32 (2.07 , N/A) | NA | 0.0921 |
| Male | 43 | 27 | 7.83 (4.77 , 20.62) | 0.34 (0.2 , 0.59) |  | 32 | 4.41 (2.96 , 9.28) | 0.18 (0.08 , 0.39) |  |
| **Ascites** |  |  |  |  |  |  |  |  |  |
| None | 12 | 8 | 7.76 (3.98 , N/A) | 0.13 (0.02 , 0.8) | 0.0114 | 8 | 4.41 (3.29 , N/A) | 0.13 (0.02 , 0.83) |  |
| Slight | 22 | 13 | 11.78 (5.2 , N/A) | 0.5 (0.3 , 0.83) |  | 15 | 7.63 (4.18 , N/A) | 0.27 (0.12 , 0.61) |  |
| Moderate | 21 | 15 | 3.32 (2.11 , N/A) | 0.18 (0.06 , 0.57) |  | 19 | 2.11 (1.91 , 4.77) | NA | 0.0026 |
| **Evidence of cirrhosis** |  |  |  |  |  |  |  |  |  |
| No | 9 | 7 | 7.07 (2.96 , N/A) | 0.15 (0.03 , 0.92) | 0.7771 | 8 | 4.93 (2.57 , N/A) | 0.13 (0.02 , 0.79) | 0.7683 |
| Yes | 42 | 29 | 5.2 (4.18 , 19.57) | 0.3 (0.18 , 0.53) |  | 33 | 4.05 (3.29 , 9.28) | 0.13 (0.06 , 0.33) |  |
| **Major Hepatic vein Portal vein Invasion** |  |  |  |  |  |  |  |  |  |
| No | 22 | 11 | 19.97 (7.07 , N/A) | 0.54 (0.32 , 0.9) | 0.0028 | 14 | 9.28 (4.54 , N/A) | 0.3 (0.14 , 0.67) | 0.0012 |
| Yes | 32 | 25 | 4.18 (3.32 , 7.83) | 0.13 (0.05 , 0.37) |  | 28 | 2.96 (2.07 , 4.41) | 0.04 (0.01 , 0.25) |  |
| **Vascular involvement** |  |  |  |  |  |  |  |  |  |
| No | 22 | 11 | 19.97 (7.07 , N/A) | 0.54 (0.32 , 0.9) | 0.0028 | 14 | 9.28 (4.54 , N/A) | 0.3 (0.14 , 0.67) | 0.0012 |
| Yes | 32 | 25 | 4.18 (3.32 , 7.83) | 0.13 (0.05 , 0.37) |  | 28 | 2.96 (2.07 , 4.41) | 0.04 (0.01 , 0.25) |  |
| **Portal vein thrombosis** |  |  |  |  |  |  |  |  |  |
| No | 22 | 11 | 19.97 (7.07 , N/A) | 0.54 (0.32 , 0.9) | 0.0028 | 14 | 9.28 (4.54 , N/A) | 0.3 (0.14 , 0.67) | 0.0012 |
| Yes | 32 | 25 | 4.18 (3.32 , 7.83) | 0.13 (0.05 , 0.37) |  | 28 | 2.96 (2.07 , 4.41) | 0.04 (0.01 , 0.25) |  |
| **Tumor nodularity** |  |  |  |  |  |  |  |  |  |
| Multinodular | 41 | 23 | 7.83 (4.41 , 21.58) | 0.43 (0.27 , 0.67) | 0.1496 | 29 | 4.05 (2.96 , 9.8) | 0.17 (0.07 , 0.4) | 0.3966 |
| Uninodular | 14 | 13 | 6.71 (2.47 , N/A) | 0.08 (0.01 , 0.51) |  | 13 | 4.18 (1.97 , N/A) | 0.08 (0.01 , 0.51) |  |
| **Metastasis** |  |  |  |  |  |  |  |  |  |
| No | 35 | 22 | 7.76 (5.2 , 20.62) | 0.29 (0.15 , 0.56) | 0.2813 | 26 | 4.41 (3.29 , 9.28) | 0.17 (0.07 , 0.4) | 0.4767 |
| Yes | 20 | 14 | 4.18 (2.47 , N/A) | 0.28 (0.12 , 0.68) |  | 16 | 3.82 (2.07 , N/A) | 0.08 (0.01 , 0.5) |  |
| **Lymph node metastasis** |  |  |  |  |  |  |  |  |  |
| No | 30 | 20 | 7.07 (4.18 , N/A) | 0.32 (0.17 , 0.61) | 0.2825 | 23 | 5.2 (3.98 , 10.26) | 0.23 (0.11 , 0.49) | 0.0395 |
| Yes | 25 | 16 | 6.71 (3.82 , 19.97) | 0.23 (0.09 , 0.59) |  | 19 | 2.96 (2.07 , 7.07) | NA |  |

Abbreviations: N, number; E, event(death); E*, event(PD or death); CTP, Child-Turcotte-Pugh; IGF, insulin-like growth factor-1; N/A, not applicable; OS, overall survival; PFS, progression-free survival.
