# Supplementary material for: Insulin-like growth factor 1/Child-Turcotte-Pugh composite score as a predictor of treatment outcomes in patients with advanced hepatocellular carcinoma treated with sorafenib
Source: Oncotarget. 2021 Apr 13;12(8):756–66. doi: 10.18632/oncotarget.27924 (PMC8057275; doi:10.18632/oncotarget.27924)
Supplement: Supplementary file 5 [file oncotarget-12-756-s005.docx]

**Supplementary Table 4: Adverse events in HCC patients with CTP class A**

|  |  | **IGF/CTP reclassification** | |  |
| --- | --- | --- | --- | --- |
| **Adverse event** | **Levels** | **AA**  ***n* (%)** | **AB**  ***n* (%)** | **Fisher exact test *p* value** |
| Fatigue | All grades | 29 (67.4) | 14 (66.7) | 0.7001 |
|  | Grade I-II | 20 (46.5) | 8 (38.1) |  |
|  | Grade III-IV | 9 (20.9) | 6 (28.6) | . |
|  | Unknown | 14 (32.6) | 7 (33.3) | . |
| Weight loss | All grades | 4 (9.4) | 2 (9.5) | 0.6359 |
|  | Grade I-II | 2 (4.7) | 2 (9.5) |  |
|  | Grade III-IV | 2 (4.7) | 0 (0) | . |
|  | Unknown | 39 (90.7) | 19 (90.5) | . |
| Anorexia | All grades | 6 (13.9) | 4 (20) | 0.4485 |
|  | Grade I-II | 5 (11.6) | 2 (10) |  |
|  | Grade III-IV | 1 (2.3) | 2 (10) | . |
|  | Unknown | 37 (86) | 16 (80) | . |
| Nausea | All grades | 12 (27.9) | 4 (19) | 0.5474 |
|  | Grade I-II | 12 (27.9) | 4 (19) |  |
|  | Unknown | 31 (72.1) | 17 (81) |  |
| Vomiting | All grades | 6 (14.3) | 4 (19.1) | 1 |
|  | Grade I-II | 5 (11.9) | 3 (14.3) |  |
|  | Grade III-IV | 1 (2.4) | 1 (4.8) |  |
|  | Unknown | 36 (85.7) | 17 (81) |  |
| Diarrhea | All grades | 12 (27.9) | 5 (23.8) | 1 |
|  | Grade I-II | 8 (18.6) | 4 (19) |  |
|  | Grade III-IV | 4 (9.3) | 1 (4.8) |  |
|  | Unknown | 31 (72.1) | 16 (76.2) |  |
| Constipation | All grades | 8 (18.6) | 2 (9.5) | 0.8019 |
|  | Grade I-II | 7 (16.3) | 2 (9.5) |  |
|  | Grade III-IV | 1 (2.3) | 0 (0) |  |
|  | Unknown | 35 (81.4) | 19 (90.5) |  |
| Rash | All grades | 8 (18.6) | 1 (4.8) | 0.3839 |
|  | Grade I-II | 4 (9.3) | 1 (4.8) |  |
|  | Grade III-IV | 4 (9.3) | 0 (0) |  |
|  | Unknown | 35 (81.4) | 20 (95.2) |  |
| Hand and foot syndrome | All grades | 8 (18.6) | 1 (4.8) | 0.4427 |
|  | Grade I-II | 3 (7) | 0 (0) |  |
|  | Grade III-IV | 5 (11.6) | 1 (4.8) |  |
|  | Unknown | 35 (81.4) | 20 (95.2) |  |
| Upper GI hemorrhage | All grades | 1 (2.3) | 1 (4.8) | 1 |
|  | Grade III-IV | 1 (2.3) | 1 (4.8) |  |
|  | Unknown | 42 (97.7) | 20 (95.2) | . |
| Lower GI hemorrhage | All grades | 1 (2.3) | 1 (4.8) | 1 |
|  | Grade III-IV | 1 (2.3) | 1 (4.8) |  |
|  | Unknown | 42 (97.7) | 2 0(95.2) | . |
| Nose bleed | All grades | 1 (2.3) | 1 (4.8) | 1 |
|  | Grade I-II | 1 (2.3) | 1 (4.8) |  |
|  | Unknown | 42 (97.7) | 20 (95.2) | . |
| Elevated transaminases | All grades | 31 (72.1) | 19 (90.5) | 0.194 |
|  | Grade I-II | 29 (67.4) | 17 (81) |  |
|  | Grade III-IV | 2 (4.7) | 2 (9.5) |  |
|  | Unknown | 12 (27.9) | 2 (9.5) |  |
| Hyperbilirubinemia | All grades | 22 (51.2) | 16 (76.2) | 0.1085 |
|  | Grade I-II | 20 (46.5) | 13 (61.9) |  |
|  | Grade III-IV | 2 (4.7) | 3 (14.3) |  |
|  | Unknown | 21 (48.8) | 5 (23.8) |  |
| Hypomagnesemia | All grades | 18 (41.8) | 7 (36.8) | 1 |
|  | Grade I-II | 17 (39.5) | 7 (36.8) |  |
|  | Grade III-IV | 1 (2.3) | 0 (0) | . |
|  | Unknown | 25 (58.1) | 12 (63.2) | . |
| Thrombocytopenia | All grades | 8 (18.6) | 11 (52.4) | 0.0087 |
|  | Grade I-II | 8 (18.6) | 11 (52.4) |  |
|  | Unknown | 35 (81.4) | 10 (47.6) | . |
| Low Hemoglobin level | All grades | 18 (41.9) | 9 (42.9) | 0.5156 |
|  | Grade I-II | 18 (41.9) | 8 (38.1) |  |
|  | Grade III-IV | 0 (0) | 1 (4.8) | . |
|  | Unknown | 25 (58.1) | 1 2(57.1) | . |
| Hypertension | All grades | 4 (9.3) | 2 (9.6) | 1 |
|  | Grade I-II | 1 (2.3) | 1 (4.8) |  |
|  | Grade III-IV | 3 (7) | 1 (4.8) | . |
|  | Unknown | 39 (90.7) | 19 (90.5) | . |
| Encephalopathy | All grades | 2 (4.6) | 2 (9.5) | 0.4999 |
|  | Grade I-II | 1 (2.3) | 0 (0) |  |
|  | Grade III-IV | 1 (2.3) | 2 (9.5) | . |
|  | Unknown | 41 (95.3) | 19 (90.5) | . |
| Lower-extremity ulcer from venous Insufficiency | All grades | 2 (4.6) | 0 (0) | 1 |
|  | Grade I-II | 1 (2.3) | 0 (0) | . |
|  | Grade III-IV | 1 (2.3) | 0(0%) | . |
|  | Unknown | 41 (95.3) | 21 (100) |  |
| Vaginal bleeding | All grades | 2 | 0 | 1 |
|  | Grade I-II | 1 (2.3) | 0 (0) |  |
|  | Grade III-IV | 1 (2.3) | 0 (0) | . |
|  | Unknown | 41 (95.3) | 2 1(100) | . |
| Esophageal varices | All grades | 2 (4.6) | 0 (0) | 1 |
|  | Grade I-II | 1 (2.3) | 0 (0) |  |
|  | Grade III-IV | 1 (2.3) | 0 (0) | . |
|  | Unknown | 41 (95.3) | 21 (100) | . |
| Renal failure secondary to hepatorenal syndrome | All grades | 2 (4.6) | 1 (4.8) | 1 |
|  | Grade I-II | 1 (2.3) | 0 (0) |  |
|  | Grade III-IV | 1 (2.3) | 1 (4.8) | . |
|  | Unknown | 41 (95.3) | 20 (95.2) | . |
| Keratoacanthoma | All grades | 2 (4.6) | 0 (0) | 1 |
|  | Grade I-II | 1 (2.3) | 0 (0) | . |
|  | Grade III-IV | 1 (2.3) | 0 (0) | . |
|  | Unknown | 41 (95.3) | 21 (100) |  |
| Liver failure | All grades | 0 (0) | 1 (4.8) | 0.3281 |
|  | Grade III-IV | 0 (0) | 1 (4.8) |  |
|  | Unknown | 43 (100) | 20 (95.2) | . |

Abbreviations: CTP, Child-Turcotte-Pugh; GI, gastrointestinal; HCC, hepatocellular carcinoma; IGF, insulin-like growth factor-1.
